# Perioperative enhanced recovery after surgery protocols with anterior mediastinal ectopic parathyroid tumor: A case report

**DOI:** 10.1097/MD.0000000000050333

**Published:** 2026-08-21

**Authors:** Xiaodan Zhu, Yingxia Yu, Kanghui Huang

**Affiliations:** a Department of Nursing, The Fourth Affiliated Hospital of School of Medicine, and International School of Medicine, International Institutes of Medicine, ZheJiang University, YiWu, China.

**Keywords:** anterior mediastinal ectopic parathyroid tumor, case report, enhanced recovery after surgery, nursing

## Abstract

**Rationale:**

To summarize the perioperative nursing experience in the successful treatment of anterior mediastinal ectopic parathyroid tumors.

**Patient concerns:**

A 50-year-old female, who had previously undergone treatment for a parathyroid adenoma for 7 years, was admitted to the hospital because of recurrent pancreatitis, kidney stones, hypercalcemia (2.98 mmol/L), elevated parathyroid hormone levels (299 pg/mL), and an abnormal parathyroid gland discovered in an external hospital examination 2 months prior.

**Diagnoses:**

Primary hyperparathyroidism secondary to an anterior mediastinal ectopic parathyroid tumor.

**Interventions:**

After the patient was admitted to the hospital, an multidisciplinary team discussion was immediately organized and a multidisciplinary collaborative team consisting of endocrinology, radiology, thoracic surgery, general surgery, and nursing departments was formed. The multidisciplinary team evaluated the patient’s treatment and ultimately decided to perform pre-diaphragmatic tumor resection under ^99^mTcO_4_-MIBI localization and developed a perioperative rapid recovery plan for the patient.

**Outcomes:**

The patient achieved rapid recovery and was discharged 2 days after surgery. At discharge, serum parathyroid hormone decreased to 16 pg/mL, and serum calcium decreased to 2.68 mmol/L.A follow-up intervention was conducted half a year after discharge, and the patient remained in good recovery condition.

**Lessons:**

A multidisciplinary enhanced recovery after surgery protocol with a clinical decision support system monitoring enables safe ultra-early discharge, addresses the unique challenges of anterior mediastinal ectopic parathyroid tumor surgery, and provides a replicable framework for complex endocrine perioperative care.

## 1. Introduction

Primary hyperparathyroidism is characterized by hypercalcemia caused by excessive secretion of parathyroid hormone (PTH), with a prevalence of approximately 1 to 7 cases per 1000 adults.^[1]^ Surgical resection of abnormal parathyroid tissue remains a radical treatment with a high success rate in typical cervical cases.^[2,3]^ However, primary hyperparathyroidism presents significant challenges when pathogenic parathyroid tissue is ectopic. Such lesions are often difficult to detect using conventional surgical exploration and increase the risk of persistent or recurrent disease.^[4]^ Ectopic parathyroid adenomas, accounting for approximately 20% of all parathyroid adenoma cases, are an important cause of refractory and recurrent hyperparathyroidism.^[4,5]^ Because of their concealed anatomical location adjacent to vital organs such as the thymus, aorta, and trachea, perioperative management of parathyroid adenomas is challenging. Surgery may cause extensive soft tissue damage, prolonged postoperative recovery, and increased risks of complications such as hypocalcemia, recurrent laryngeal nerve injury, and mediastinal infection, which can lead to prolonged hospital stay.^[5]^

Enhanced recovery after surgery (ERAS),^[6]^ a multimodal perioperative care protocol aimed at minimizing surgical stress, optimizing physiological function, and accelerating patient recovery, has been widely used in gastrointestinal, orthopedic, and thoracic surgery. The core measures of ERAS include preoperative patient education, optimized anesthesia, minimally invasive surgical techniques, early mobilization, and personalized nutritional support. These measures shorten the hospital stay, reduce postoperative complications, and improve patient satisfaction.^[5]^ However, current research on ERAS is predominantly focused on common surgical procedures, and data on the use of ERAS in cases of ectopic anterior mediastinal parathyroid tumor resection are limited.

In a systematic review, Chorath et al^[7]^ reported that ERAS protocols in parathyroid surgery primarily focused on cervical cases, and hospital stay was significantly reduced compared with traditional care. Nevertheless, anterior mediastinal ectopic cases involve distinct surgical approaches, such as video-assisted thoracoscopic surgery or open thoracotomy, and unique perioperative challenges, such as mediastinal drainage management and the maintenance of respiratory function. However, these approaches differ significantly from those used for cervical surgery. To date, only sporadic case reports have described surgical techniques for treating anterior mediastinal ectopic parathyroid tumors,^[4]^ and studies on ERAS-based perioperative nursing are lacking.

Herein, we present the case of a patient with an anterior mediastinal ectopic parathyroid tumor who underwent surgical resection combined with perioperative ERAS. The treatment involved multidisciplinary collaboration, monitoring and management of serum calcium and PTH levels, personalized care-oriented psychological nursing, dynamic pain management, and early pulmonary rehabilitation exercises. The patient recovered rapidly and was successfully discharged on the second postoperative day. To the best of our knowledge, this is the first systematic summary of ERAS nursing strategies for treating anterior mediastinal ectopic parathyroid tumors. Our findings may provide practical and standardized perioperative nursing guidance for the clinical management of such complex anterior mediastinal ectopic parathyroid tumor cases.

## 2. Case presentation

A 50-year-old woman was admitted to our department with the main complaint of “more than 7 years after parathyroid adenoma surgery.” Seven years prior, the patient had developed abdominal pain accompanied by nausea and vomiting without obvious inducement. The patient was diagnosed with pancreatitis at a different hospital. Further examination suggested that pancreatitis was caused by hyperparathyroidism. The patient underwent a left thyroid resection, left parathyroidectomy, and exploration of the right thyroid lobe. Pathological examination confirmed parathyroid adenoma. After surgery, she received cinacalcet orally for treatment and underwent regular reexaminations; however, she stopped the medication on her own. During this period, the patient developed recurrent pancreatitis, kidney stones, and other diseases. The ectopic parathyroid gland was found more than 2 months ago, and pancreatitis recurred 2 month ago. The patient was transferred to our department for further treatment.

Upon admission, auxiliary examination revealed that the left thyroid had been resected, and no obvious abnormalities were found in the bilateral parathyroid regions. Baseline respiratory function and mobility were normal. The patient had no chronic pulmonary disease, dyspnea, or limited physical activity before surgery. The PTH concentration was 299 pg/mL and the blood calcium was 2.98 mmol/L. Localization imaging with ^99^mTc-MIBI suggested an anterior mediastinal ectopic parathyroid tumor.

We systematically completed differential diagnosis and clinical reasoning to confirm the diagnosis of recurrent primary hyperparathyroidism caused by anterior mediastinal ectopic parathyroid adenoma. First, we excluded secondary hyperparathyroidism induced by chronic renal insufficiency, vitamin D deficiency, and malabsorption syndrome via serum creatinine, vitamin D, and intestinal function tests. Second, we ruled out malignant parathyroid carcinoma: the patient had no rapidly progressive hypercalcemia, palpable cervical mass, or elevated serum alkaline phosphatase, and imaging showed a single well-demarcated anterior mediastinal lesion without invasive signs. Third, thoracic lymphoma, thymoma, and mediastinal metastatic tumors were excluded by enhanced chest computed tomography combined with nuclear medicine imaging. Combined with the patient’s history of prior parathyroid surgery, recurrent hypercalcemia and recurrent pancreatitis, we confirmed the pathogenic lesion was an ectopic parathyroid tumor located in the anterior mediastinum.

We organized a multidisciplinary consultation, including specialists from the Departments of Endocrinology, Thoracic Surgery, General Surgery, Radiology, Nuclear Medicine, Anesthesiology, and a nursing team. We formulated a comprehensive treatment plan, including video‑assisted thoracoscopic surgery (VATS) and perioperative ERAS nursing.

The patient underwent VATS resection of the anterior mediastinal ectopic parathyroid tumor. A multidisciplinary team (MDT) managed the patient. The serum calcium and PTH levels were monitored, and the patient underwent care-oriented personalized psychological nursing, dynamic pain management, and early pulmonary rehabilitation exercises.

All serum calcium and PTH test results were automatically uploaded to the hospital clinical decision support system (CDSS) platform immediately after laboratory reporting. After data entry, the system completed automatic model matching, risk stratification, and intervention recommendation output within approximately 15 minutes for this patient. Specifically, the system first captured the patient’s baseline hypercalcemia data on admission, triggered a high-risk alert, and pushed a 4-hour interval monitoring plan; after intraoperative PTH sharp decline, the system rapidly identified hypoparathyroidism risk and reminded nurses to prepare oral calcium supplements in advance.

**Figure 1.**
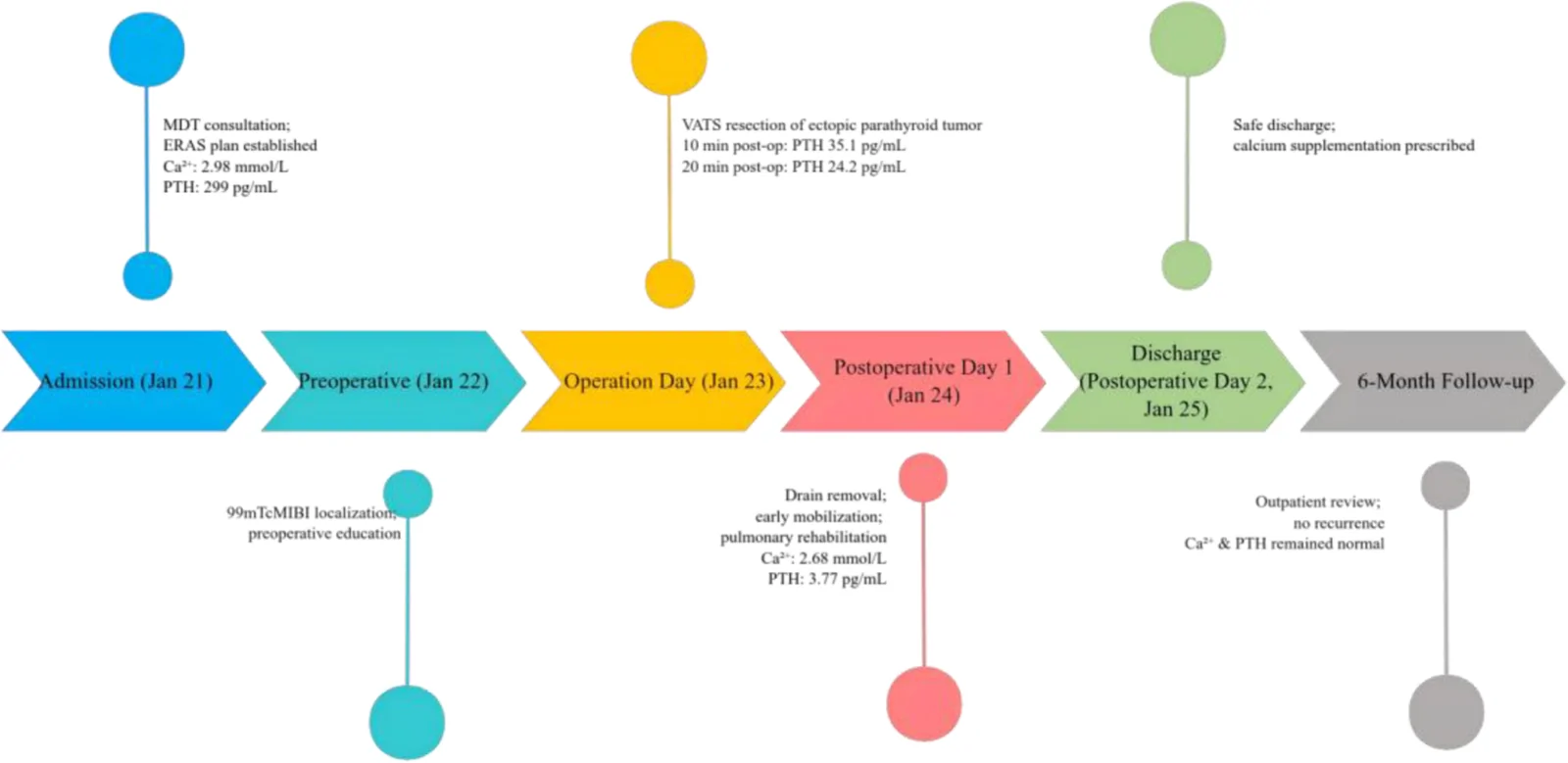
Visual timeline of the patient’s clinical course. ERAS = enhanced recovery after surgery, MDT = multidisciplinary team, PTH = parathyroid hormone, VATS = video-assisted thoracoscopic surgery.

The patient’s condition stabilized postoperatively. Both the blood calcium and PTH concentrations improved significantly, and the patient was discharged from the hospital 2 days after the operation.The patient’s clinical timeline is presented in Figure 1.

## 3. Nursing care

### 3.1. Multidisciplinary collaboration

To address the complexity of the perioperative management of this patient with an anterior mediastinal ectopic parathyroid tumor, we created a MDT composed of specialists from endocrinology, radiology, thoracic surgery, general surgery, and nursing departments. The core objective of this team was to integrate the concept of ERAS to optimize the nursing process. Endocrinology specialists participated in the comprehensive evaluation of primary hyperparathyroidism, including monitoring of serum calcium and PTH levels, to clarify the severity of the disease and guide preoperative metabolic regulation. Radiologists precisely localized ectopic tumors using imaging examinations, such as contrast-enhanced neck computed tomography and ^99^mTcO_4_-MIBI parathyroid scintigraphy^[8]^ providing a crucial basis for surgical planning. Considering the anatomical complexity of the anterior mediastinum, physicians in thoracic and general surgery jointly discussed the VATS approach to minimize intraoperative trauma. Nursing staff participated in assessing patients’ physical and psychological status. Nutritionists evaluated baseline nutritional status to assess the risk of malnutrition, which may exacerbate surgical stress. This collaborative process resulted in a personalized perioperative nursing plan tailored to the patient’s specific conditions, with the goals of reducing perioperative stress, minimizing complications, and accelerating postoperative recovery. The collaboration ensured seamless communication between the various disciplines, effectively integrating medical, surgical, and nursing expertise within the ERAS framework and providing a foundation for optimized perioperative care.

### 3.2. Serial monitoring of serum calcium and parathyroid hormone

Given the preoperative suspicion of hyperparathyroidism, we postoperatively monitored the serum calcium and PTH levels to track the evolution of parathyroid function and guide calcium metabolism management.^[9]^ A CDSS^[10]^ was integrated into this monitoring framework to enhance the precision and timeliness of interventions. Real-time serial monitoring data were integrated with the patients’ baseline characteristics, such as the preoperative calcium concentration and surgical approach. Monitoring was performed using a predefined algorithm that mapped PTH/calcium trends to evidence-based action thresholds. The monitoring data processed by the CDSS revealed characteristic changes.

Admission day (1.21): serum calcium, 2.98 mmol/L; PTH, 299 pg/mL, consistent with the laboratory manifestations of hyperparathyroidism. At this point, the CDSS flagged a “high-risk” status for hypercalcemic complications and recommended increasing the monitoring frequency every 4 hours.

Post-lesion resection (1.23): 10 minutes after lesion excision, PTH plummeted to 35.1 pg/mL and further decreased to 24.2 pg/mL at 20 minutes post-resection. This change in PTH level triggered the CDSS to issue an early alert for impending hypoparathyroidism, prompting the nursing team to prepare for oral calcium supplementation.

**Figure 2.**
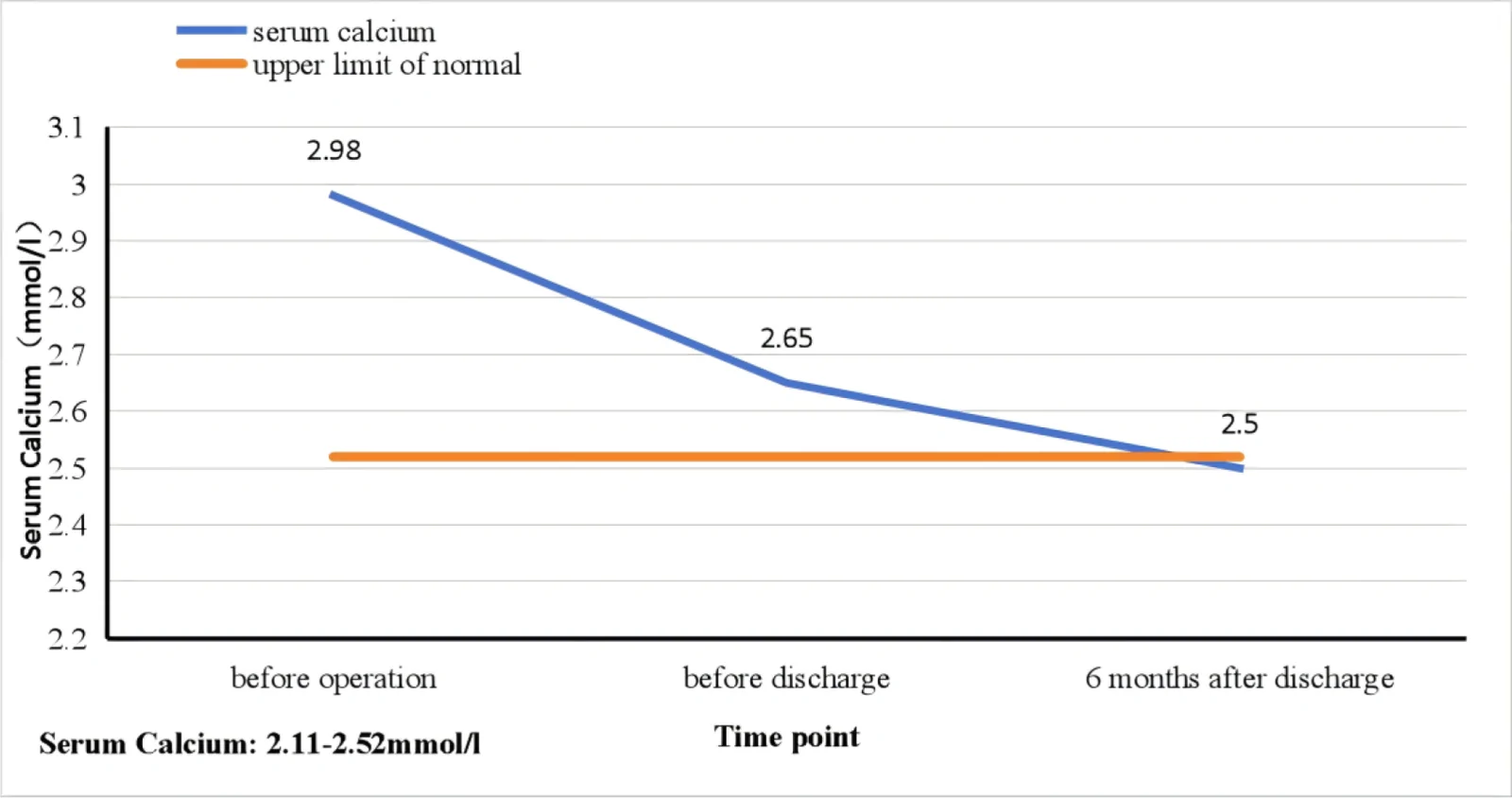
Trends in serum calcium concentration throughout hospitalization and follow-up.

**Figure 3.**
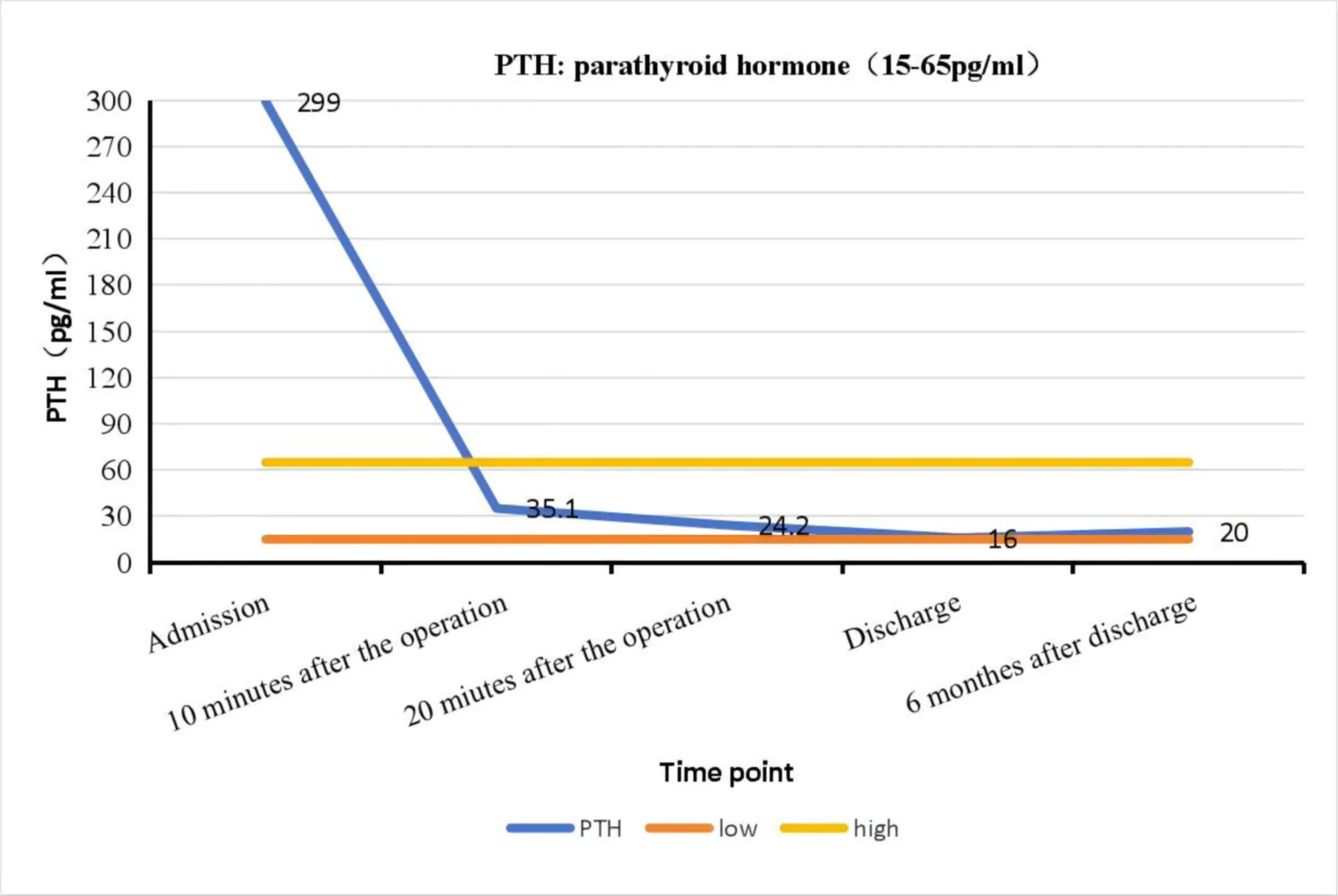
Dynamic changes of serum parathyroid hormone levels in patients during the perioperative period. PTH = parathyroid hormone.

Predischarge (1.24): serum calcium declined to 2.68 mmol/L, as illustrated in Figure 2, and PTH dropped to 16 pg/mL, with dynamic changes in PTH presented in Figure 3. By cross-referencing these values with trends on postoperative day 1, the CDSS generated a tailored calcium supplementation protocol of 500 mg twice daily, with weekly PTH rechecks.

CDSS provided data-driven suggestions within approximately 15 minutes of data entry, supporting timely recognition of calcium dynamics and providing auxiliary data reference and personalized calcium supplementation. Complications such as tetany and muscle spasms caused by hypocalcemia were prevented by adjusting the timing and dose of calcium supplementation based on PTH concentration. Moreover, synergy ensures the safety of early pulmonary rehabilitation exercises and ambulation, embodying the core logic of “precise monitoring-targeted intervention” in ERAS, with CDSS providing auxiliary data support for clinical decision-making.

### 3.3. Early pulmonary rehabilitation exercises

Early pulmonary rehabilitation is an essential component of the ERAS protocol. Early rehabilitation is vital for patients undergoing anterior mediastinal surgery. Surgical trauma, postoperative pain, and immobility may compromise respiratory function and increase the risk of atelectasis, pneumonia, and respiratory insufficiency.^[11]^ Therefore, we implemented a structured, phased pulmonary rehabilitation program to promote lung expansion, strengthen respiratory muscles, and prevent pulmonary complications, with close integration of clinical assessments to guide progression. The program was delivered in a stepwise manner, tailored to the patient’s recovery trajectory (Table 1) .

**Table 1:** Summary of staged pulmonary rehabilitation program.

Phase	Time	Intervention	Frequency	Goal
Immediate	0–6 h	Diaphragmatic breathing	q2h	Prevent atelectasis
Early	6–24 h	Spirometry + guided cough	q3h	Improve ventilation
Subacute	24–48 h	Ambulation + deep breathing	Tolerance-based	Enhance recovery

1. Immediate postoperative period, 0 to 6 hours after awakening. Continuous monitoring of vital signs and oxygen saturation. Nurses guided the patient through gentle diaphragmatic breathing exercises (5–8 breaths/min, sustained for 5 minutes, repeated every 2 hours). This low-intensity intervention was intended to mitigate alveolar collapse without causing excessive pain or fatigue. Concurrently, regular position adjustments every 2 hours from supine to 30° semi-Fowler’s position were performed to optimize lung ventilation and facilitate mucus drainage.
2. Early postoperative period (6–24 hours): with pain effectively controlled (VAS ≤ 3), exercise intensity was gradually increased. The patient was instructed to use a spirometer, performing 10 repetitions per session, with each inhalation sustained for 3 to 5 seconds, conducted every 3 hours. To clear airway secretions and prevent mucus retention, guided and effective coughing exercises were introduced, with the patient supporting the incision with both hands to minimize pain during coughing. Posteroanterior chest radiography was performed 24 hours postoperatively. The patient had good pulmonary re-expansion with no signs of atelectasis, pneumothorax, or pleural effusion, consistent with the effectiveness of early rehabilitation.^[12]^ Considering the stable respiratory status and minimal drainage volume (<50 mL in the preceding 8 hours), we removed the mediastinal drain under aseptic conditions. This milestone not only reduced the physical discomfort associated with the drain but also enhanced the patient’s willingness to engage in subsequent activities.
3. Subacute postoperative period (24–48 hours): following drain removal, we integrated pulmonary rehabilitation with early mobilization. The patient was assisted to sit at the bedside for 5 to 10 minutes, followed by standing and slow ambulation, initially 5 m, gradually extended to 10 to 15 meters, while performing deep breathing. This synergistic approach improved lung ventilation, promoted diaphragmatic movement, and enhanced overall respiratory efficiency.^[13]^ Throughout the process, nurses continuously monitored the respiratory rate, oxygen saturation, and breath sounds. Nurses adjusted exercise intensity based on tolerance, for example, by reducing the duration of dyspnea or increasing pain. Patient education was reinforced to emphasize adherence: “Each deep breath helps your lungs expand fully, lowering infection risk and speeding your return to daily life.” This structured early pulmonary rehabilitation program coupled with timely radiological assessment and drain removal effectively maintains adequate pulmonary ventilation. Postoperatively, the patient’s oxygen saturation remained stable at 98% to 100% on room air, with no respiratory complications.

### 3.4. Dynamic pain management

Dynamic pain management, a cornerstone of ERAS,^[14]^ was implemented to mitigate postoperative pain and facilitate early rehabilitation with scheduled flurbiprofen administration (Q12H) as the core intervention. Six hours postoperatively, flurbiprofen (100 mg orally) was administered every 12 hours to establish preemptive analgesia, with a target pain score (VAS) of ≤3 and movement-related pain, for example, during pulmonary rehabilitation, ≤4. Pain was assessed every 4 hours, with additional evaluations before mobilization or respiratory training. If breakthrough pain (VAS score > 3) occurred, rescue analgesia (e.g., 50 mg tramadol) was administered, and the nursing team adjusted non-pharmacological measures, such as optimizing the semi-Fowler’s position (30° elevation) during coughing or applying cold compresses to the surgical site, to enhance efficacy. This multimodal strategy, combining scheduled flurbiprofen (Q12H) administration with real-time pain monitoring, provided consistent analgesia. The patient’s average VAS remained at 2.1 (rest) and 3.2 (movement) during hospitalization, which avoided pain-induced respiratory suppression or delayed ambulation.

### 3.5. Psychological nursing

Centered on humane care, personalized perioperative psychological nursing is tailored to patients’ emotional needs.^[15]^ Preoperatively, nurses used in-depth interviews to identify stressors such as concerns about surgical outcomes and anxiety over postoperative recovery. Nurses built trust through empathetic communication, e.g., “It’s normal to worry about recovery; we’ll adjust the plan based on how you feel.” Therefore, postoperative intervention should be considered. Because of the patient’s introverted personality, we provided a “mood diary” to help the patient express unspoken concerns, and her emotional experiences were validated. Family support was integrated by guiding relatives to use supportive communication, and the patients’ favorite music playlists were curated to reduce their unfamiliarity with the environment. When the patient’s anxiety intensified during tube removal, the nurses used process visualization, explained the steps using hand-drawn diagrams, and held the patient’s hand during the procedure to convey a sense of security. This approach improved patient satisfaction and enhanced adherence to rehabilitation training. By prioritizing individual experiences over standardized protocols, nurses conveyed a sense of security and embodied humane care to facilitate holistic recovery.

### 3.6. Discharge guidance and follow-up

This study obtained formal ethical approval from the Ethics Committee of the Fourth Affiliated Hospital, Zhejiang University School of Medicine (Approval No. K2025229). Written informed consent was fully signed and obtained from the patient prior to clinical data collection and manuscript preparation.

To support the patient’s rapid recovery and safe transition to home care, discharge guidance was streamlined around key self-management priorities, while continuous care was provided by “Internet +” teleconsultation.^[16]^ Upon discharge on postoperative day 2, personalized guidance was focused on key matters such as the recognition of hypocalcemia symptoms (paresthesia and muscle cramps) and a progressive rehabilitation plan (30 minutes of daily walking, avoiding weight-bearing). A printed checklist with illustrations, including symptom comparison charts and medication schedules, was provided to enhance adherence. The core of the follow-up was an online consultation platform through which patients could access 24/7 text and image consultation services from a nursing team. For urgent situations, such as persistent numbness, remote video links to a MDT (endocrinologists and surgeons) ensured timely assessment within 2 hours. This model addresses the post-discharge safety gaps. Readmission was avoided through remote consultation. By bridging in-hospital and home care via digital tools, the goal of accelerated recovery was sustained while ensuring ongoing safety, with 100% patient satisfaction reported in follow-up feedback.

## 4. Discussion

This case report describes the successful application of a multidisciplinary ERAS protocol integrated with a CDSS in a patient with an anterior mediastinal ectopic parathyroid tumor. To our knowledge, this is one of the few reports focusing on systematic perioperative nursing care using an ERAS strategy for this rare condition. The patient achieved rapid recovery and was safely discharged on postoperative day 2, with sustained biochemical and clinical stability during the 6-month follow-up period. These outcomes support the feasibility and safety of a tailored ERAS program in complex endocrine mediastinal surgery.

Previous studies on mediastinal ectopic parathyroid adenomas have mainly focused on surgical techniques, imaging localization, and short-term postoperative outcomes. For example, Hemead et al^[4]^ reported a case of ectopic mediastinal parathyroid adenoma and emphasized the value of accurate localization and complete surgical resection. However, their report did not describe a structured perioperative rehabilitation program, nor did it involve dynamic monitoring of PTH and calcium or early mobilization strategies. In contrast, the present case highlights a comprehensive ERAS protocol that combines multidisciplinary collaboration, metabolic monitoring, early pulmonary rehabilitation, dynamic analgesia, and psychological support. The present study therefore extends the literature by focusing not only on surgical success but also on nursing‑centered accelerated recovery, which is critical for early discharge and reduced complication risk.

The ERAS protocol used in this study differs from standard thoracic ERAS programs in several important ways. Conventional thoracic ERAS primarily emphasizes pulmonary care, pain control, fluid management, and early mobilization for lung, esophageal, or mediastinal mass surgeries. In comparison, the protocol applied here was customized to address the unique pathophysiology of primary hyperparathyroidism and ectopic parathyroid tumor resection. Key differences include continuous dynamic monitoring of PTH and calcium, CDSS-assisted early warning of hypocalcemia, personalized calcium supplementation strategies, and tighter collaboration with endocrinology and nuclear medicine teams. These additions ensure metabolic stability, prevent life‑threatening electrolyte disturbances, and create a safer foundation for early mobilization and pulmonary rehabilitation. Thus, the current protocol represents a disease‑specific adaptation of ERAS rather than a direct application of general thoracic surgery ERAS.

This study has several limitations that should be acknowledged. First, it is a single case report without a control group, which limits the generalizability of the findings and prevents statistical comparison between the ERAS intervention and traditional care. Second, original inpatient medical records did not record the specific maximum diameter of the anterior mediastinal ectopic parathyroid tumor and detailed intraoperative gross pathological findings, which leads to an incomplete clinical baseline data description and is an inherent limitation of this case report. Third, the CDSS component was applied in a single patient and lacked external validation or comparison with conventional manual interpretation. Finally, the cost-effectiveness and resource utilization of this multidisciplinary ERAS model were not analyzed. Future studies with larger sample sizes, controlled designs, and long-term follow-sup are needed to verify the efficacy, safety, and sustainability of this protocol.

Despite these limitations, this case demonstrates that a tailored, multidisciplinary ERAS protocol supported by CDSS-guided metabolic monitoring can facilitate safe, rapid recovery and early discharge in patients undergoing surgery for anterior mediastinal ectopic parathyroid tumors. This approach may serve as a reference for perioperative nursing and multidisciplinary care in similar complex endocrine mediastinal surgeries.

## Author contributions

**Writing – original draft:** Xiaodan Zhu.

**Writing – review & editing:** Yingxia Yu, Kanghui Huang.

## References

[1] Glasgow C Lau EYC Aloj L. An approach to a patient with primary hyperparathyroidism and a suspected ectopic parathyroid adenoma. J Clin Endocrinol Metab. 2022;107:1706–13. 35150267 10.1210/clinem/dgac024

[2] Bilezikian JP Khan AA Silverberg SJ. Evaluation and management of primary hyperparathyroidism: summary statement and guidelines from the fifth international workshop. J Bone Miner Res. 2022;37:2293–314. 36245251 10.1002/jbmr.4677

[3] Kowalski GJ Buła G Żądło D Gawrychowska A Gawrychowski J. Primary hyperparathyroidism. Endokrynol Pol. 2020;71:260–70. 32797471 10.5603/EP.a2020.0028

[4] Hemead HM Abdellatif AA Rahman MAA. Ectopic pure mediastinal parathyroid adenoma: a case report. Int J Surg Case Rep. 2022;90:106598. 34896776 10.1016/j.ijscr.2021.106598 PMC8666502

[5] Rabazzi G Elia G Aprile V. Surgical management of mediastinal ectopic parathyroids. J Pers Med. 2025;15:276. 40710393 10.3390/jpm15070276 PMC12300348

[6] Ljungqvist O Scott M Fearon KC. Enhanced recovery after surgery: a review. JAMA Surg. 2017;152:292–8. 28097305 10.1001/jamasurg.2016.4952

[7] Chorath K Luu N Go BC Moreira A Rajasekaran K. ERAS protocols for thyroid and parathyroid surgery: a systematic review and meta-analysis. Otolaryngol Head Neck Surg. 2022;166:425–33. 34126805 10.1177/01945998211019671

[8] Ovčariček PP Giovanella L Gasset IC. The EANM practice guidelines for parathyroid imaging. Eur J Nucl Med Mol Imaging. 2021;48:2801–22. 33839893 10.1007/s00259-021-05334-y PMC8263421

[9] Palermo A Tabacco G Makras P. Primary hyperparathyroidism: from guidelines to outpatient clinic. Rev Endocr Metab Disord. 2024;25:875–96. 39162944 10.1007/s11154-024-09899-5

[10] Olakotan O Yusof MM Puteh SEW. A systematic review on CDSS Alert Appropriateness. Stud Health Technol Inform. 2020;270:906–10. 32570513 10.3233/SHTI200293

[11] Tang Z Lu M Qu C. Enhanced recovery after surgery improves short-term outcomes in patients undergoing esophagectomy. Ann Thorac Surg. 2022;114:1197–204. 34624264 10.1016/j.athoracsur.2021.08.073

[12] Medbery RL Fernandez FG Khullar OV. ERAS and patient reported outcomes in thoracic surgery: a review of current data. J Thorac Dis. 2019;11(Suppl 7):S976–86. 31183180 10.21037/jtd.2019.04.08 PMC6535471

[13] Batchelor TJP Rasburn NJ Abdelnour-Berchtold E. Guidelines for enhanced recovery after lung surgery: recommendations of the Enhanced Recovery After Surgery (ERAS®) Society and the European Society of Thoracic Surgeons (ESTS). Eur J Cardiothorac Surg. 2019;55:91–115. 30304509 10.1093/ejcts/ezy301

[14] Gottumukkala V Joshi GP. Challenges and opportunities in enhanced recovery after surgery programs: an overview. Indian J Anaesth. 2024;68:951–8. 39659530 10.4103/ija.ija_546_24 PMC11626874

[15] Lv J Su Y Tang H Jiang X Chen X. Humanistic nursing care management strategies: from formulation to implementation. Front Public Health. 2025;13:1591077. 40421357 10.3389/fpubh.2025.1591077 PMC12104067

[16] Zhang GW Li B Gu ZM. In-depth examination of the functionality and performance of the internet hospital information platform: development and usability study. J Med Internet Res. 2024;26:e54018. 39168813 10.2196/54018 PMC11584526

